# Relationship between selenium intake, circulating selenium levels, and stroke: a systematic review and meta-analysis

**DOI:** 10.3389/fneur.2025.1578103

**Published:** 2025-12-04

**Authors:** Yanyan Li, Rui Ding, Xiaorui Pei, Peng Gao, Ziqi Liu, Lifeng Piao

**Affiliations:** 1 Department of Neurology Ward, Chaoyang Central Hospital of China Medical University, Chaoyang, Liaoning, China; 2 Department of Molecular Biology Laboratory, Chaoyang Central Hospital of China Medical University, Chaoyang, Liaoning, China; 3 Department of Reproductive Medicine, Chaoyang Central Hospital of China Medical University, Chaoyang, Liaoning, China; 4 Xinhua Clinical College, Dalian University, Dalian, Liaoning, China; 5 Department of Respiratory Ward, Chaoyang Central Hospital of China Medical University, Chaoyang, Liaoning, China

**Keywords:** nutrition, stroke, selenium, microelements, antioxidants

## Abstract

**Background:**

Ischemic stroke represents the most prevalent form of cerebrovascular disease, which has a significant impact on people’s quality of life. Selenium is a crucial trace mineral with potential relevance for the prevention of cerebrovascular disease due to its antioxidant properties. Recent research has increasingly linked circulating selenium levels to the incidence of ischemic stroke, however, the findings remained inconsistent. The primary objective of our meta-analysis is to explore the potential relationship between circulating selenium levels and stroke as well as stroke mortality. In the meantime, the current study was done to evaluate the influence of dietary selenium intake on the risk of stroke.

**Methods:**

A comprehensive systematic search of electronic databases was conducted from inception through April 11, 2025, to identify relevant studies investigating the associations between circulating selenium levels, dietary selenium intake and ischemic stroke risk. Ultimately, 26 eligible studies were included in the meta-analysis.

**Results:**

(1) The aggregated weighted mean difference (WMD) demonstrated that circulating selenium concentrations were markedly reduced in the ischemic stroke cohort relative to the control cohort (WMD = −0.13 [−0.20, −0.07]). (2) The multivariable-adjusted relative risk (RR) indicated that increased circulating selenium levels linked to a notable decrease in the risk of ischemic stroke (RR = 0.88 [0.83,0.92]), as well as a decreased risk of stroke mortality (RR = 0.86 [0.80, 0.93]). (3) Furthermore, our meta-analysis found that increased dietary selenium intake was adversely correlated with the risk of stroke, with RR of 0.87(0.76, 0.99). (4) A meta-analysis of dose–response curves revealed that circulating selenium levels were adversely linked with stroke.

**Conclusion:**

The level of circulation selenium is lower in ischemic stroke patients. There was an inverse association between the level of circulation selenium and the incidence of ischemic stroke as well as stroke mortality. Meanwhile, higher dietary selenium intake were shown to be negatively associated with ischemic stroke.

## Introduction

To our knowledge, stroke is globally recognized as the third major contributor to disability and the second leading cause of death, resulting in increasing socioeconomic burdens (1, 2). According to current forecasts, by 2030, there will be a 20.5% rise in the prevalence of strokes compared to 2012 (2). Reducing stroke risk is an urgent priority, and identifying modifiable risk factors is essential for prevention and treatment. Over 90% of strokes worldwide are linked to controllable factors, including cardio-metabolic diseases, smoking, and unhealthy dietary habits (3–5). Consequently, the identification of novel risk factors are essential for reducing stroke risk. Among potential modifiable factors, nutritional determinants have emerged as increasingly prominent targets for stroke prevention.

The pathophysiology of ischemic stroke involves multiple interconnected pathways, such as inflammation, oxidative stress, and ionic imbalances. Current evidence particularly highlights oxidative stress as a central mediator in stroke pathophysiology (6). Selenium (Se) is a vital trace element with antioxidant capabilities for humans and is a natural dietary component (7), serving as an important regulator of brain function. It is believed that the antioxidant capabilities of selenium-dependent glutathione peroxidases (GPxs) provide cardio protection (8, 9). The neuroprotective action of Se may be achieved through controlling of selenium-containing antioxidants, cell signaling pathways and activation of transcription factors (10–13). However, results from experimental and observational investigations on the correlation between circulating selenium concentrations and incidence and mortality of cerebrovascular disorders were inconsistent (14–18). Some investigations have revealed that selenium is negatively correlated with the incidence of stroke (15, 16). Notably, some studies have failed to demonstrate a clinically meaningful association between elevated selenium levels and decreased stroke risk (14, 18).

Consequently, numerous studies (14–18), including systematic reviews, have investigated the relationship between selenium and cerebrovascular diseases. A systematic review by Kuria et al. (19) demonstrated that higher circulating selenium levels were associated with reduced incidence and mortality from cardiovascular disease compared to lower levels, confirming its protective role. Their study also revealed a statistically significant non-linear dose-response relationship between selenium levels and cardiovascular disease mortality. Although this evidence established a link with cardiovascular outcomes, it did not specifically address the association with stroke. In 2021, Ding and Zhang (20) published a systematic review incorporating 12 observational studies, which revealed an inverse correlation between circulating selenium levels and stroke. This association was further corroborated in cross-sectional/case–control studies and specifically within investigations measuring whole blood selenium. Although this review established a link with circulating selenium, it did not perform a dose–response analysis. Furthermore, it focused exclusively on circulating biomarkers and did not investigate the relationship between dietary selenium intake and stroke. Although previous meta-analyses have linked circulating selenium to stroke, key limitations persist. Firstly, the relationship between dietary selenium intake (a modifiable risk factor) and stroke lacks conclusive evidence and has not been systematically assessed in a meta-analysis. Secondly, the recent publication of numerous new cohort studies justifies an updated and more robust analysis. Lastly, previous reviews have not addressed dose–response relationships. To bridge these gaps, this study aims to: (1) quantify the association between dietary selenium and stroke risk; (2) investigate the dose–response relationship; and (3) compare these findings with evidence on circulating selenium.

## Materials

### Search strategy and study selection

**Figure 1:**
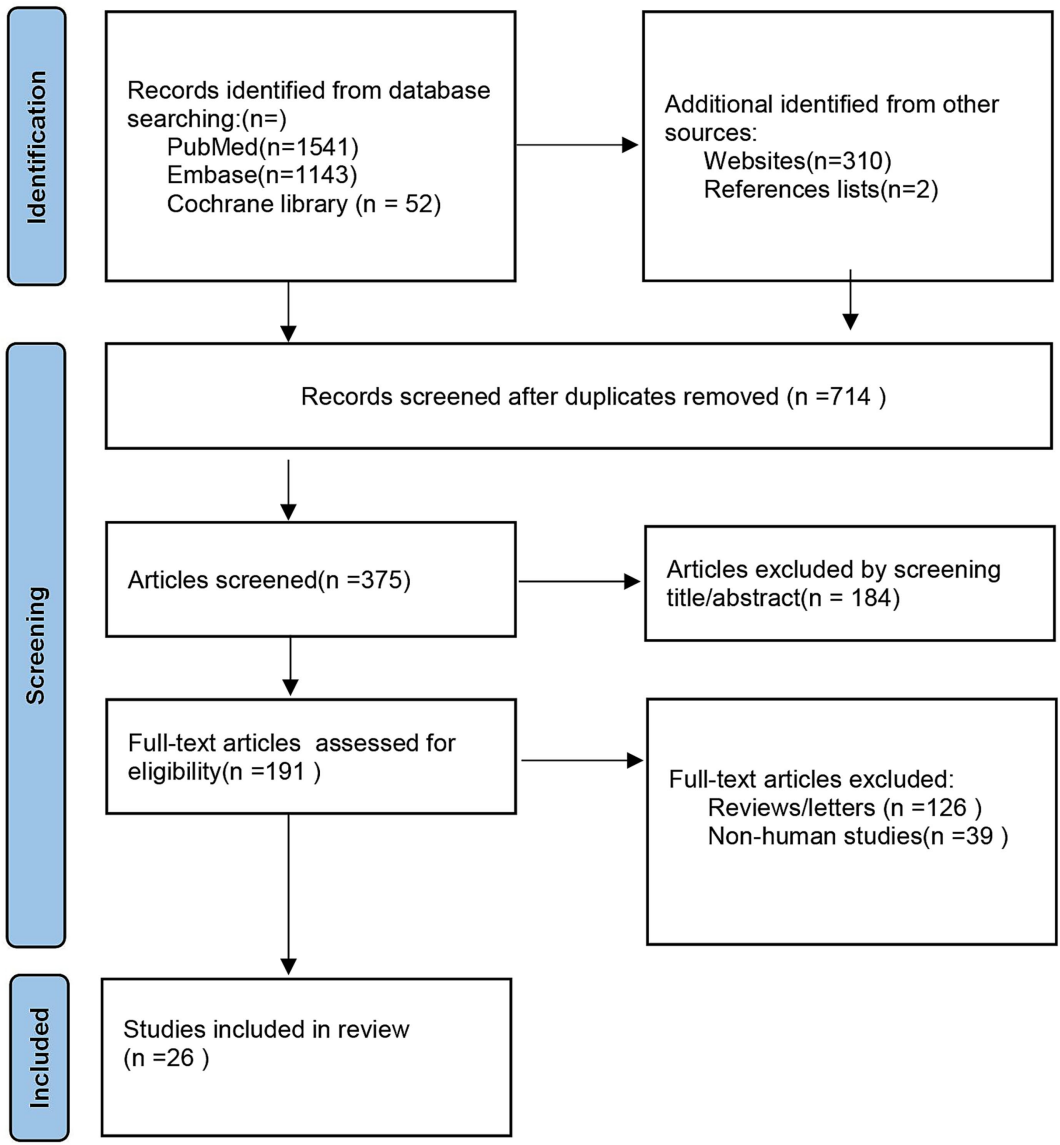
Preferred Reporting Item for Systematic Reviews and Meta-Analysis (PRISMA) guideline.

A thorough literature search was carried out from the beginning to April 11, 2025, using MEDLINE, PubMed, the Cochrane Library, Web of Science, and the EMBASE databases. Additional records were acquired from Google Scholar’s, reference list of included studies and potentially related articles, which were not detected during the literature search. In the search technique, the following search phrases and keywords were linked by “and” or “or”: ischemic stroke, cerebral infarction, cerebrovascular illness, selenium, trace elements, and microelements. Two writers independently chose publications from the resulting list, screening the article titles and abstracts. The articles that passed the screening were thoroughly reviewed in full text based on the eligibility criteria. The study was done using the Preferred Reporting Item for Systematic Reviews and Meta-Analysis (PRISMA) standard (21) (Figure 1).

## Methods

### Data extraction and study quality

#### Inclusion criteria

The search methodology was confined to publicly accessible data and articles in the English language. Publications were selected according to the following criteria: (1) The studies were prospective cohort studies and case–control studies; (2) they examined the relationship between selenium and cerebral infarction, ischemic stroke, or cerebrovascular disease; (3) they provided relative risk (RR), odds ratio (OR), or weighted mean difference (WMD) with a 95% confidence interval (CI). The research investigating the correlation between dietary selenium and stroke necessitated the following criteria: (1) The research was either a prospective cohort study or a case–control study; (2) it assessed the correlation between dietary selenium consumption and stroke risk; (3) the primary outcome was stroke; and (4) the study presented relative risk estimates or odds ratios with a 95% confidence interval in case–control studies.

#### Exclusion criteria

Two reviewers independently retrieved data from pertinent articles utilizing data extraction forms that encompassed details such as authorship, publication year, research design, sample size, various biomarker levels, and patient outcomes. We excluded: (1) duplicate or irrelevant papers; (2) reviews, letters, or case reports; (3) non-original research (editorials, reviews, or commentaries); and (4) studies involving non-human subjects.

#### Data extraction

The gathered information includes the first author, publication year, location, age, gender, sample size, study methodology, exposure category, effect estimates, and modifications. The impact estimates corrected for the maximum number of confounding factors, together with their 95% confidence intervals (CIs), for the comparison between the highest and lowest circulating selenium levels, were obtained. Additionally, the circulating selenium levels (mean ± standard deviation [SD]) were obtained for both stroke and control patients. Conversely, the unit of circulating selenium was standardized to “umol/L” throughout all trials. Research on dietary selenium and stroke evaluated the following elements: methods for assessing selenium intake, relative risk (RR) and related confidence intervals (CI) for stroke at maximum and minimum levels, and the factors controlled in the research. All studies reporting whole blood selenium were cross-sectional in nature. In all cohort studies, serum/plasma selenium served as the exposure variable. The assessment methods for selenium intake included in the study are shown in Table 1. Consequently, sensitivity analysis was performed for cross-sectional/case–control studies and for serum/plasma selenium investigations, respectively.

**Table 1:** Characteristics of studies included in this meta-analysis.

Study and year	Country	Study type	*N* total	Case (umol/L)	Control (umol/L)	RR (stroke or death)	Se biomarker	Quality-NOS
Selenium and stroke								
Hu et al. (2017) (35)	Canada	Cross-sectional	2,077	3.29 ± 0.13	4.04 ± 0.03	0.17 (0.06, 0.48)	Whole blood	9
Angelova et al. (2008) (29)	Bulgaria	Case–control	85	1.3 ± 0.5	1.7 ± 0.5	NA	Serum	8
Koyama et al. (2009) (32)	Japan	Case–control	60	1.33 ± 0.25	1.47 ± 0.21	0.28 (0.10, 0.85)	Whole blood	7
Hu CHMS et al. (2019) (44)	Canada	Prospective	7,065	2.29 ± 0.03	2.49 ± 0.01	0.38 (0.15, 0.92)	Whole blood	9
Hu NHANES et al. (2019) (44)	Canada	Prospective	5,030	2.29 ± 0.03	2.44 ± 0.01	0.57 (0.13, 1.03)	Whole blood	9
Nahan et al. (2017) (30)	USA	Case–control	75	2.38 ± 0.18	2.63 ± 0.11	NA	Plasma	6
Skalny et al. (2017) (33)	Russia	Case–control	60	1.43 ± 0.05	1.24 ± 0.07	NA	Serum	7
Wen et al. (2019) (46)	China	Case–control	2,554	1.03 ± 0.05	1.23 ± 0.06	0.10 (0.06, 0.17)	Plasma	7
Schomburg et al. (2019) (43)	Germany	Prospective	4,366	–	–	1.57 (1.21, 2.02)	Plasma	8
						1.51 (1.32, 1.72)	Plasma	
Xiao et al. (2019) (36)	China	Prospective	29,763	–	–	0.74 (0.50, 1.10)	Plasma	8
Mirończuk et al. (2021) (28)	Poland	Case–control	210	0.73 ± 0.06	0.96 ± 0.06	NA	Serum	8
Hu et al. (2021) (16)	China	Case–control	2,510	1.1 ± 0.24	1.11 ± 0.24	0.78 (0.6, 0.99)	Plasma	7
Fang et al. (2022) (18)	China	Case–control	9,639	–	–	0.968 (0.914, 1.026)	Serum	8
Wang et al. (2022) (15)	China	Case–control	1,236	1.06 ± 0.25	1.08 ± 0.26	0.50 (0.31, 0.80)	Plasma	8
Wang et al. (2022) (31)	China	Case–control	3,808	0.83 ± 0.04	0.84 ± 0.04	0.86 (0.76, 0.96)	Plasma	8
Kok et al. (1987) (34)	Netherlands	Case–control	10,532	1.46 ± 0.06	1.6 ± 0.01	0.5 (0.2, 1.25)	Serum	7
						3.2 (0.8, 12.1)	Serum	
Marniemi et al. (2005) (14)	Finland	Cohort	755	1.93 ± 0.03	1.83 ± 0.01	1.66 (0.87, 3.17)	Serum	9
Chang et al. (1998) (17)	Taiwan	Case–control	57	2.7 ± 0.13	2.9 ± 0.14	NA	Plasma	7
Wei et al. (2004) (48)	China	Prospective	1,103	NA	NA	0.99 (0.88, 1.11)	Serum	8
Ray et al. (2006) (47)	USA	Cohort	632	1.43 ± 0.02	1.54 ± 0.01	0.71 (0.56, 0.90)	Serum	6
Shi et al. (2022) (37)	China	Cohort	6,155	NA	NA	0.68 (0.56, 0.83)	Plasma	8
Dietary selenium intake					ug/d		Method of dietary assessment of selenium intake	
Zhang et al. (2023) (57)	China	Cohort	11,532	Quartile 2	29.9–38.53	0.85 (0.59, 1.21)	Three consecutive 24-h dietary recalls were used to assess dietary intake of participating individuals. Food consumption data were converted into the dietary selenium intake using Chinese Food Composition Tables (FCTs)	
				Quartile 3	38.53–47.23	0.62 (0.42, 0.92)		
				Quartile 4	47.23–60.38	0.43 (0.28, 0.68)		
				Quartile 5	>60.38	0.49 (0.30, 0.82)		
Shi et al. (2022) (53)	China	Cross-sectional	39,438	Quartile 2	77–108	0.70 (0.55, 0.88)	Two non consecutive days of intake data were available for each participant. Dietary selenium intake from foods was calculated using the US Department of Agriculture’s Food and Nutrient Database for Dietary Studies.	
				Quartile 3	108–148	0.71 (0.53, 0.93)		
				Quartile 4	148–400	0.61 (0.43, 0.85)		
Hu et al. (2017) (35)	Canada	Cross-sectional		Quartile 2	31–85	1.32 (0.59, 2.96)	Dietary selenium intake from country food (i.e., dietary selenium) was estimated with a food frequency questionnaire	
				Quartile 3	86–225	0.57 (0., 22, 1.48)		
				Quartile 4	226–825	0.18 (0.05, 0.71)		
Chen et al. (2024) (54)	China	Cross-sectional	26,433	–	–	1.016 (0.978, 1.055)	The diet-derived intake information was obtained from a detailed dietary interview component that estimated the types and amounts of foods and beverages consumed during the 24 h period prior to the interview	
Merrill et al. (2017) (45)	USA	Cross-sectional	27,770	Quartile 2	–	1.08 (0.87, 1.33)	Not mentioned	
				Quartile 3	–	1.21 (0.98, 1.48)		
				Quartile 4	–	1.34 (1.10, 1.64)		
Marniemi et al. (2005) (14)	USA	Case–control	2,275	–	–	0.983 (0.55, 1.77)	Derived from dietary assessment over two non-consecutive days (using 24-h dietary recall)	

#### Quality assessment

Each author independently assessed the quality and risk of bias of the included studies using the Newcastle-Ottawa score (NOS) (22); It is based on 3 broad perspectives: the selection process of study cohorts, the comparability among different cohorts, and the identification of either the exposure or outcome of study cohorts, disagreements were settled by discussion. Research that received scores of 0–4 and 5–9 were classified as high-quality and low-quality, respectively. A low NOS score typically implies that the study is of poor quality.

### Statistical analysis

The meta-analysis was conducted using STATA version 17.0 and Review Manager software version 5.3. For continuous data, mean ± standard deviation is displayed, along with the mean with standard deviation mean difference (WMD) and 95% confidence intervals (CIs). If the included studies provided the data using median and quartile values, we estimated the mean and standard deviation 17 using the Wan et al. approach (23). The data heterogeneity was assessed using I-square (I^2^) statistics. Results for included studies where I^2^ > 50% were analyzed using the random-effects model. A fixed-effect model was used if not. Statistical significance was defined as *p*-values<0.05, and 95% confidence intervals were supplied. The random effects models were used to depict odds ratios in the forest plots. A mixed logistic regression model with random treatment effects was used to determine the overall OR. The pooled effect sizes and 95% CIs were calculated using forest plots. The odds ratio (OR) was considered equivalent to the relative risk (RR). We conducted a dose–response meta-analysis utilizing the methodology established by Greenland and Longnecker (24) and Orsini et al. (25). To assess the trend from the associated log relative risk across categories of dietary selenium use. Employing this technique, we obtained the quantity of dietary selenium consumption, distributions of cases and person-years of dietary selenium consumption, distributions of cases and person-years, relative risk (RR) and 95% confidence interval (CI). The median or mean dietary selenium intake for each group was utilized as the matching open-ended, we considered the boundary equivalent to that of the next neighboring category. We assessed a possible correlation between dietary selenium and stroke risk utilizing limited cubic splines with three knots at the 10th, 50th, and 90th percentiles of the distribution. A *p*-value was determined by equating the coefficient of the second spline to zero.

A funnel plot was used to visually analyze the symmetry of the publication to determine publication bias.

## Results

### Literature search and study characteristics

A total of 3,048 citations were identified, encompassing only the titles and abstracts passed muster. Complete text of potentially relevant papers was read. Table 1 lists the features of the included researches, and Figure 1 displays the flow chart of the literature search. After removing the duplicates, 375 items were still present, a total of 191 publications underwent initial screening based on their titles and abstracts, 90 reviews, 36 case reports or letters, 39 non-human studies were removed. Eventually, the remaining 26 full-text papers were assessed based on the qualifying requirements (Figure 1).

Consequently, a total of 26 studies involving selenium and ischemic stroke, encompassing patients, were examined qualitatively, and then a meta-analysis was conducted (Table 1). Twenty one studies about the level circulating selenium and incidence of ischemic stroke with a total of 87,772 patients, 6 studies about dietary selenium intake and ischemic stroke, totaling 59,252 participants, were analyzed qualitatively and meta-analysed (Table 1). Six to nine was the range of quality scores. Every record that was included was thought to be of moderate to high quality.

### The aggregated weighted mean difference (WMD) of the circulating selenium levels between ischemic stroke patients and controls

**Figure 2:**
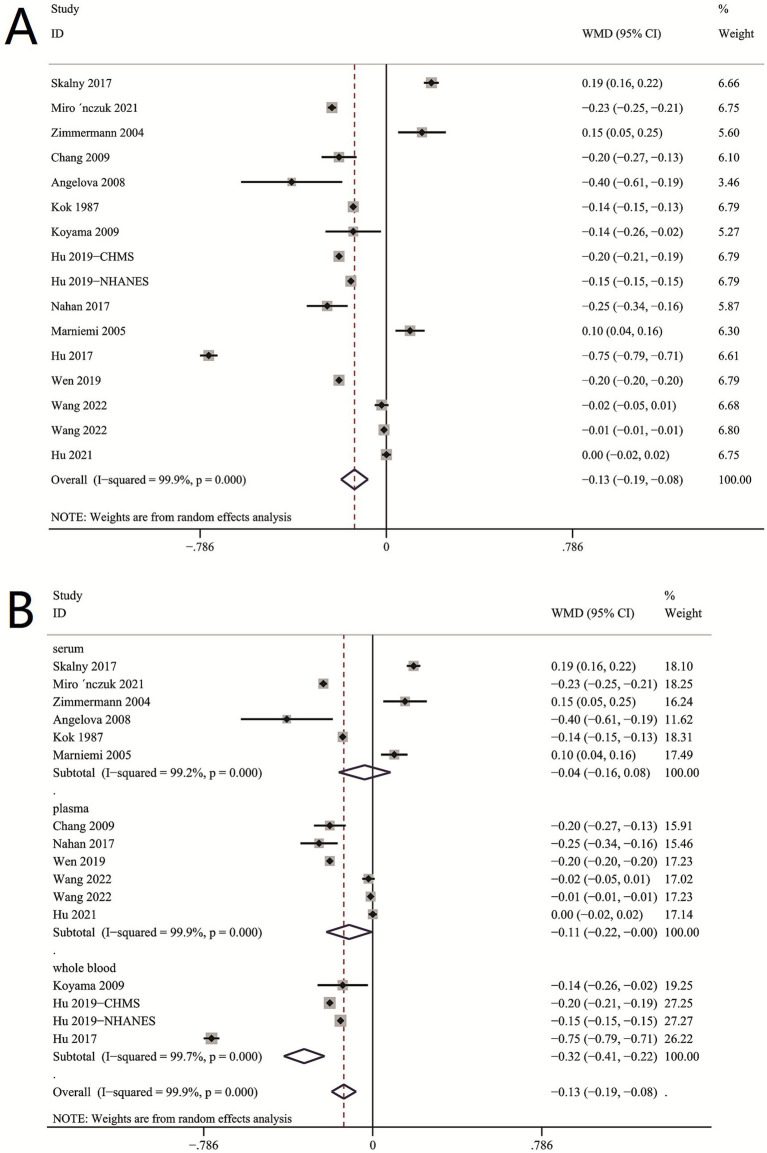
**(A)** Circulating selenium levels between ischemic stroke patients and controls. **(B)** Subgroup analysis between Se concentration between stroke patients and controls.

**Figure 3:**
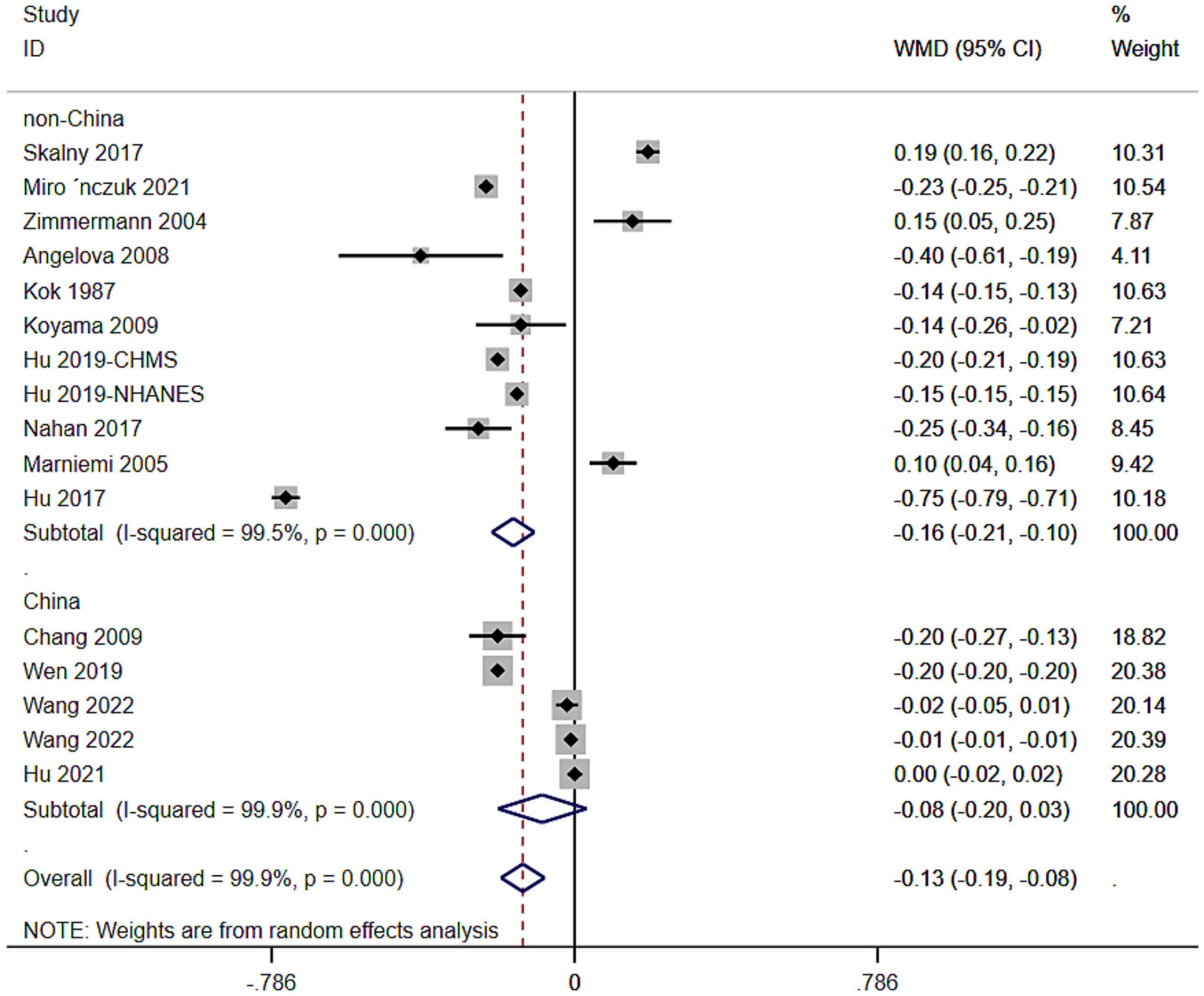
Association between circulating selenium levels and ischemic stroke risk.

The aggregated WMD showed that circulating selenium levels were lower in ischemic stroke than controls (WMD = −0.13 [−0.20,−0.07]) (Figure 2A). In subgroup analysis, we observe a difference in whole blood selenium concentration between ischemic stroke patients and controls (WMD = −0.32 [−0.41, −0.22]) (Figure 2B), but no difference in serum (WMD = −0.04 [−0.19, 0.10]) (Figure 2B) and plasma Se concentration (WMD = −0.11 [−0.21, 0.03]). According to different regions and populations, the results indicated that circulating selenium levels were lower in non-Chinese (WMD = −0.16 [−0.21, −0.10]) (Figure 3), rather than Chinese (WMD = −0.08 [−0.20, −0.07]) (Figure 3). Additionally, the results from the sensitivity analysis are presented in Table 2.

**Table 2:** Subgroup analysis of WMD and sensitivity analysis in ischemic stroke and controls.

Outcome	Studies	WMD	95%CI	I^2^ for heterogeneity	P for heterogeneity
Selenium biomarker	16				
Whole blood	4	−0.32	−0.41, −0.22	97.9%	*P* = 0.000
Serum	6	−0.04	−0.16, 0.08	99.2%	*P* = 0.000
Plasma	6	−0.11	−0.22, 0.00	94.9%	*P* = 0.000
Region	16				
Europe	5	−0.08	−0.17, 0.01	99.6%	*P* = 0.007
Asia	7	−0.05	−0.15, 0.05	99.4%	*P* = 0.000
North America	4	−0.34	−0.43, −0.24	97.1%	*P* = 0.256

### The multivariable-adjusted RR of stroke for the highest compared with the lowest categories of circulating selenium concentrations

**Figure 4:**
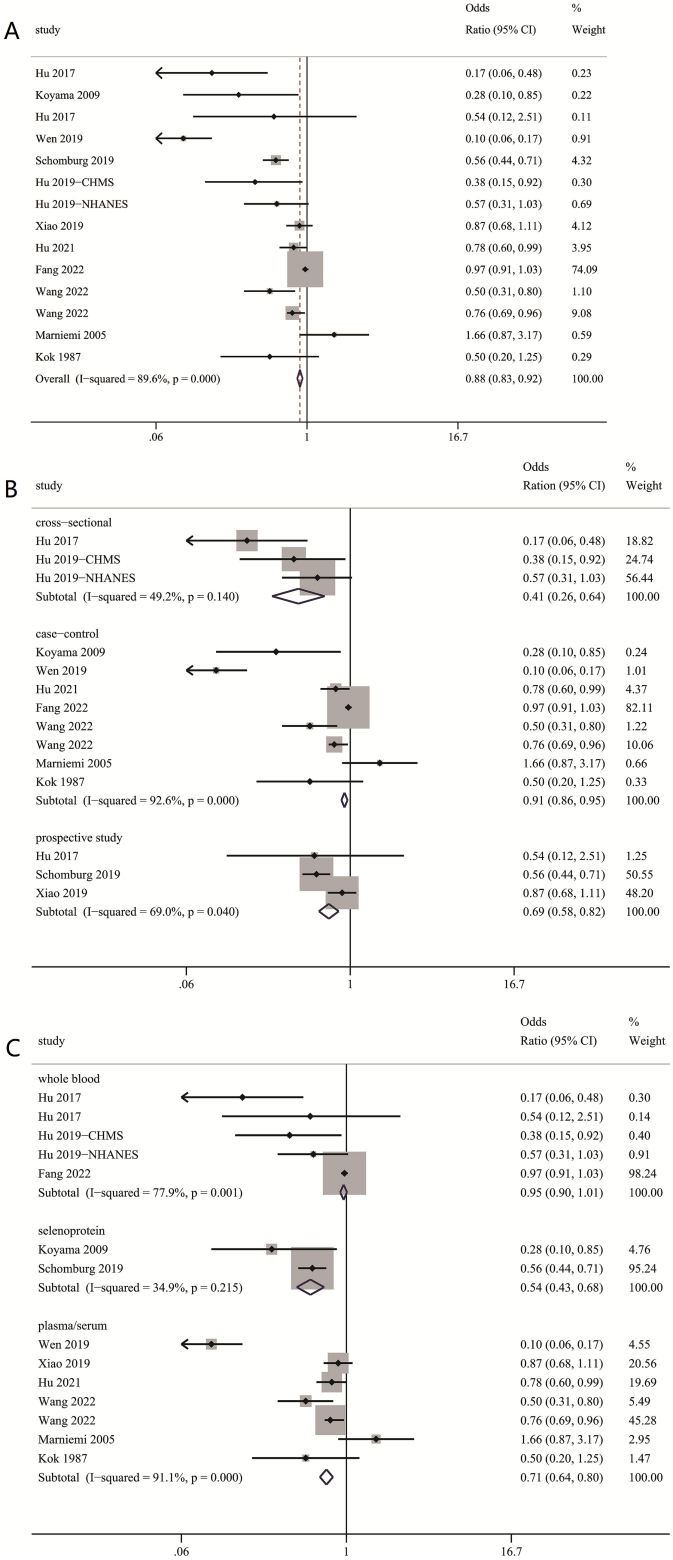
(**A**) Circulating selenium levels and ischemic stroke incidence; (**B**) Subgroup analysis Circulating selenium levels and ischemic stroke incidence by study type; (**C**) Subgroup analysis Circulating selenium levels and ischemic stroke incidence by Se biomarker.

In the present meta-analysis, the RR indicated that increased circulating selenium levels were inversely related to ischemic stroke incidence, with RR of (0.88 [0.83–0.92]) (Figure 4), rather than hemorrhagic stroke, with RR of 0.82 (0.69, 1.01) (Table 3).

**Table 3:** Subgroup and sensitivity analysis of the selenium circulating level and stroke risk.

Outcome	Studies	OR/RR	95%CI	I^2^ for heterogeneity	P for heterogeneity
Selenium biomarker	14				
Whole blood	5	0.95	0.90, 1.01	77.9%	*P* = 0.001
Serum/plasma	7	0.71	0.64, 0.80	91.1%	*P* = 0.000
Selenoprotein	2	0.54	0.43, 0.68	34.9%	*P* = 0.215
Study type	14				
Cross-sectional	3	0.41	0.26, 0.64	49.2%	*P* = 0.140
Prospective	3	0.69	0.58, 0.82	69%	*P* = 0.04
Case–control	8	0.91	0.86, 0.95	92.6%	*P* = 0.000
Sex	6				
Female	6	0.88	0.81, 0.96	76.1%	*P* = 0.032
Male	6	0.82	0.74, 0.90	59.5%	*P* = 0.679
BMI	4				
≥24	4	0.87	0.81, 0.94	57.2%	*P* = 0.03
<24	4	0.79	0.71, 0.87	67.2%	*P* = 0.027
Age	6				
≥60	6	0.84	0.77, 0.91	71.4%	*P* = 0.004
<60	6	0.83	0.75, 0.92	44.3%	*P* = 0.110
Hemorrhagic stroke	4	0.84	0.69, 1.01	41.3%	*P* = 0.164
Region	14				
Europe	3	0.63	0.51, 0.78	79.6%	*P* = 0.007
Asia	7	0.9	0.86, 0.95	93.4%	*P* = 0.000
North America	4	0.42	0.27, 0.65	26%	*P* = 0.256

### Subgroup analysis and sensitivity analysis for circulating selenium levels and ischemic stroke incidence

Significant heterogeneity was observed across studies (I^2^ = 89.6%, *p* < 0.001), consequently, we conducted subgroup analysis. There was an inverse relationship between increased circulating selenium levels and stroke incidence. The conclusion was supported by selenoprotein P (0.54 [0.43, 0.68]) (Figure 4C), plasma/serum (0.71 [0.64, 0.80]) (Figure 4C), BMI ≥ 24 (0.87 [0.81, 0.94]) (Table 3) and age≥60 years old (0.84 [0.77, 0.91]) (Table 3), case–control studies (0.91 [0.86, 0.95]) (Figure 4B), prospective studies (0.69 [0.58, 0.82]) (Figure 4B) rather than whole blood (0.95 [0.90, 1.01]) (Figure 4C) and cross-sectional studies (0.41 [0.26, 0.64]) (Figure 4B). Additionally, the results from the sensitivity analysis are presented in Table 3.

### Circulating selenium levels and stroke mortality

**Figure 5:**
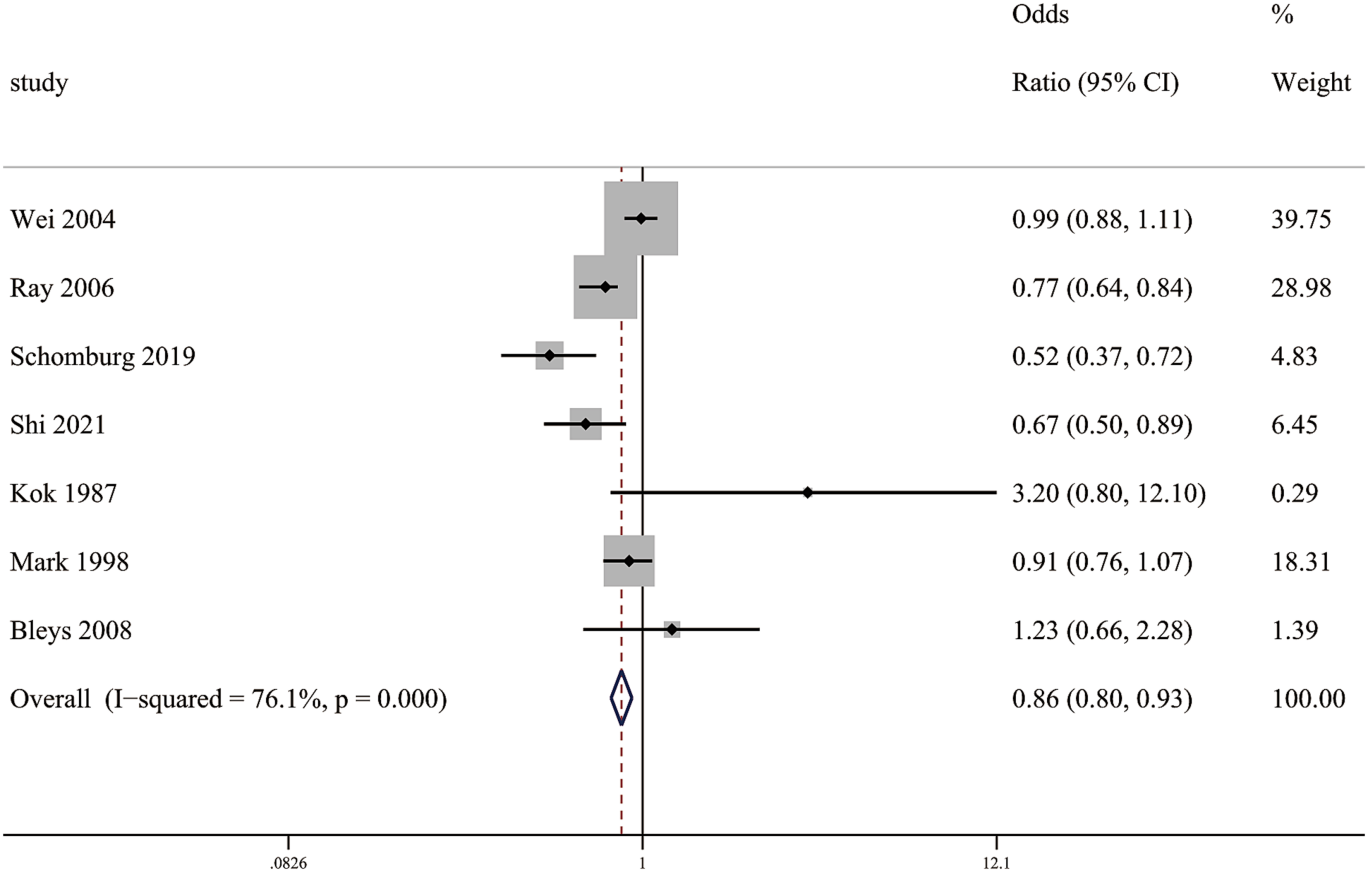
Association between circulating selenium levels and stroke mortality.

In our meta-analysis, a multivariable-adjusted RR demonstrated that elevated circulating selenium concentrations showed a significant inverse correlation with stroke mortality, with RR of (0.86 [0.80–0.93]) (Figure 5).

### The connection between the incidence of stroke and dietary selenium intake

**Figure 6:**
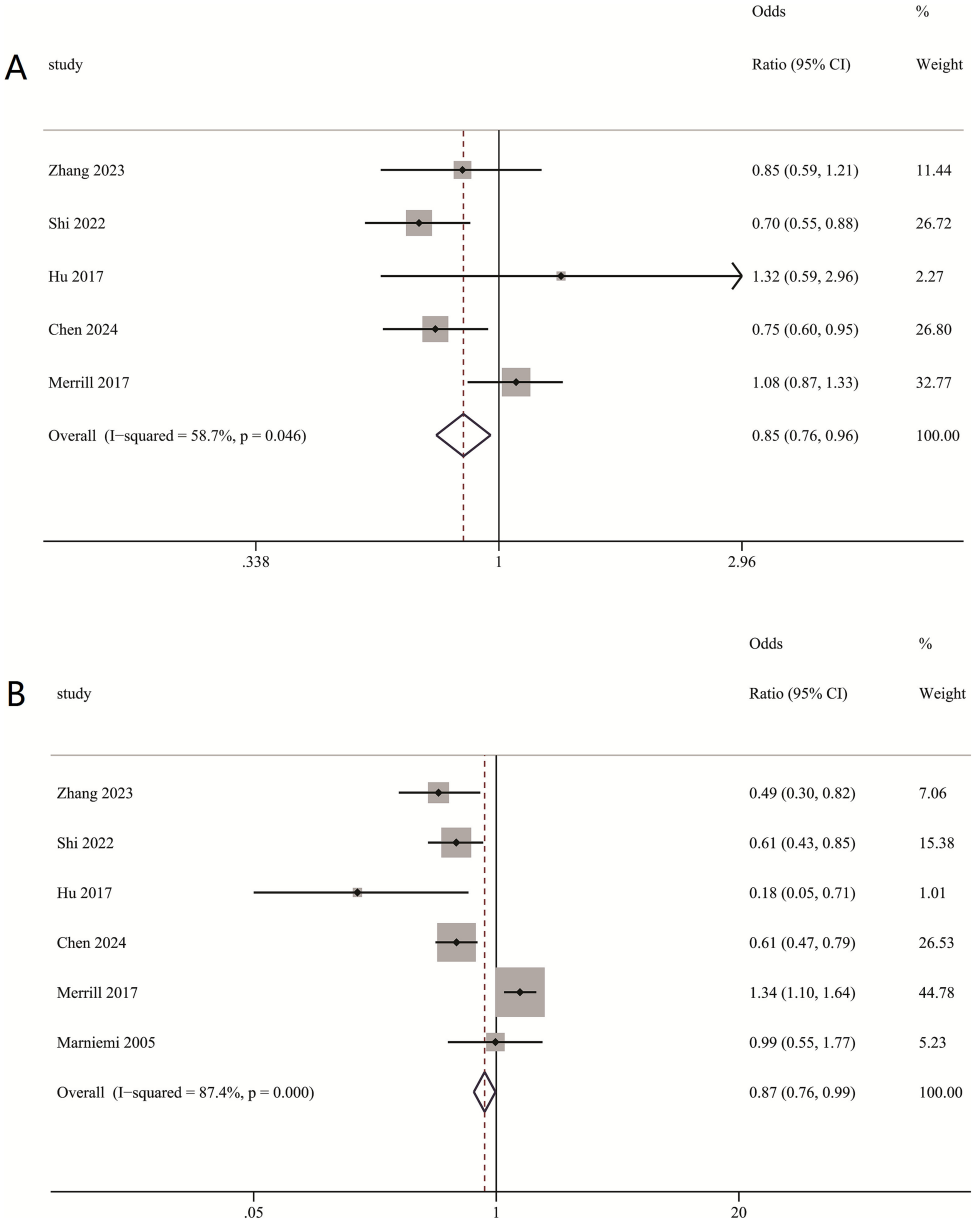
(**A**) Association between second-highest quartile dietary intake and ischemic stroke risk; (**B**) Association between highest quartile dietary intake and ischemic stroke risk.

Our meta-analysis disclosed that higher dietary selenium intake was inversely associated with ischemic stroke risk. Compared to the lowest quartile, the second-highest quartile of dietary selenium intake showed RR of 0.85 (0.76, 0.96) (Figure 6A), while the highest quartile with RR of 0.87 (0.76, 0.99) (Figure 6B).

### Subgroup and sensitivity analysis of dietary selenium intake

Subgroup analysis indicated that lower dietary selenium consumption was linked to a decreased stroke risk in females (RR = 0.91 [0.88, 0.95]), males (RR = 0.92 [0.87, 0.97]), age ≥ 60 (RR = 0.93 [0.89, 0.98]), age < 60 (RR = 0.9 [0.87, 0.99]) (Table 4). Furthermore, dietary selenium intake was substantially linked with the prevalence of ischemic stroke in hypertensive individuals (OR = 0.89 [0.85, 0.94]) (Table 4).

**Table 4:** Subgroup and sensitivity analysis of the dietary selenium intake and stroke risk.

Outcome	Studies	RR/OR	95%CI	I^2^ for heterogeneity	P for heterogeneity
Sex					
Female	4	0.91	0.88, 0.95	77.9%	*P* = 0.004
Male	4	0.92	0.87, 0.97	68.1%	*P* = 0.024
Age					
≥60	4	0.93	0.89, 0.98	77.9%	*P* = 0.004
<60	4	0.90	0.87, 0.99	71.4%	*P* = 0.015
Dietary intake level					
2 quartile	5	0.85	0.76, 0.96	58.7%	*P* = 0.046
4 quartile	5	0.87	0.73, 0.99	87.4%	*P* = 0.000
Hypertension	3	0.89	0.85, 0.94	87.1%	*P* = 0.000

### Dose–response meta-analysis

**Figure 7:**
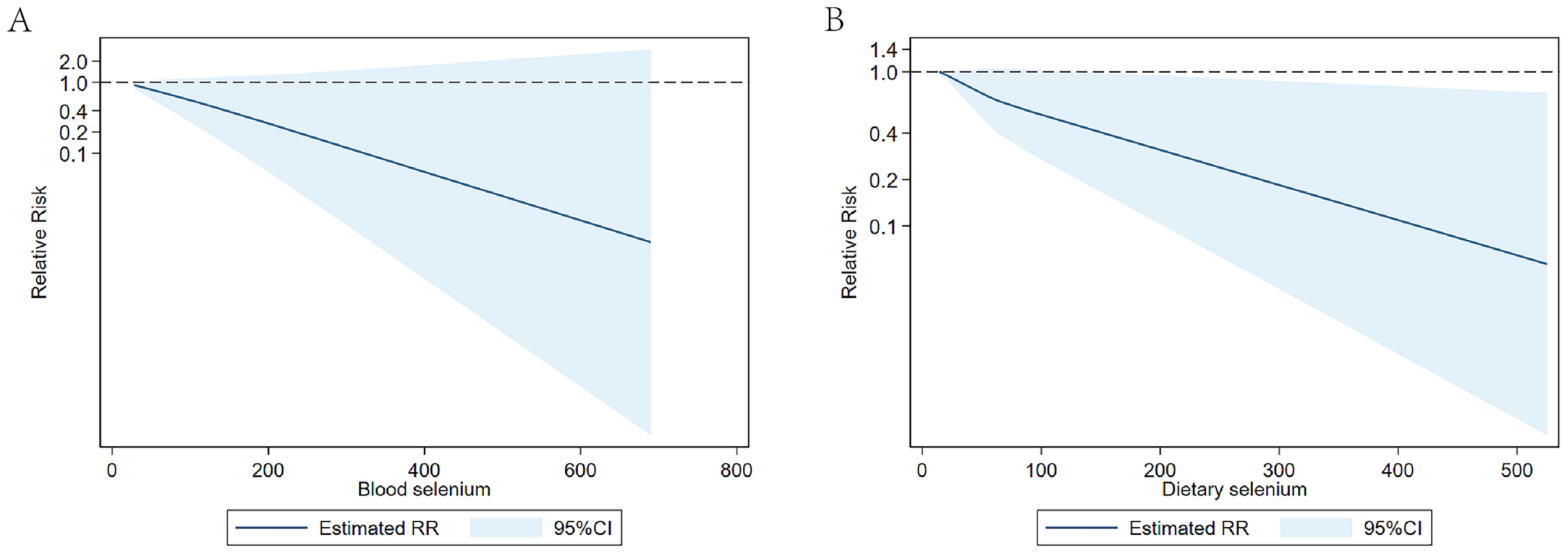
(**A**) Dose-response of circulating selenium levels and stroke risk; (**B**) Dose-response of dietary selenium intake and stroke risk.

Our analysis revealed an inverse linear relationship between circulating selenium levels and stroke risk, while no such association was observed in dietary selenium intake (Figure 7).

### Publication bias

**Figure 8:**
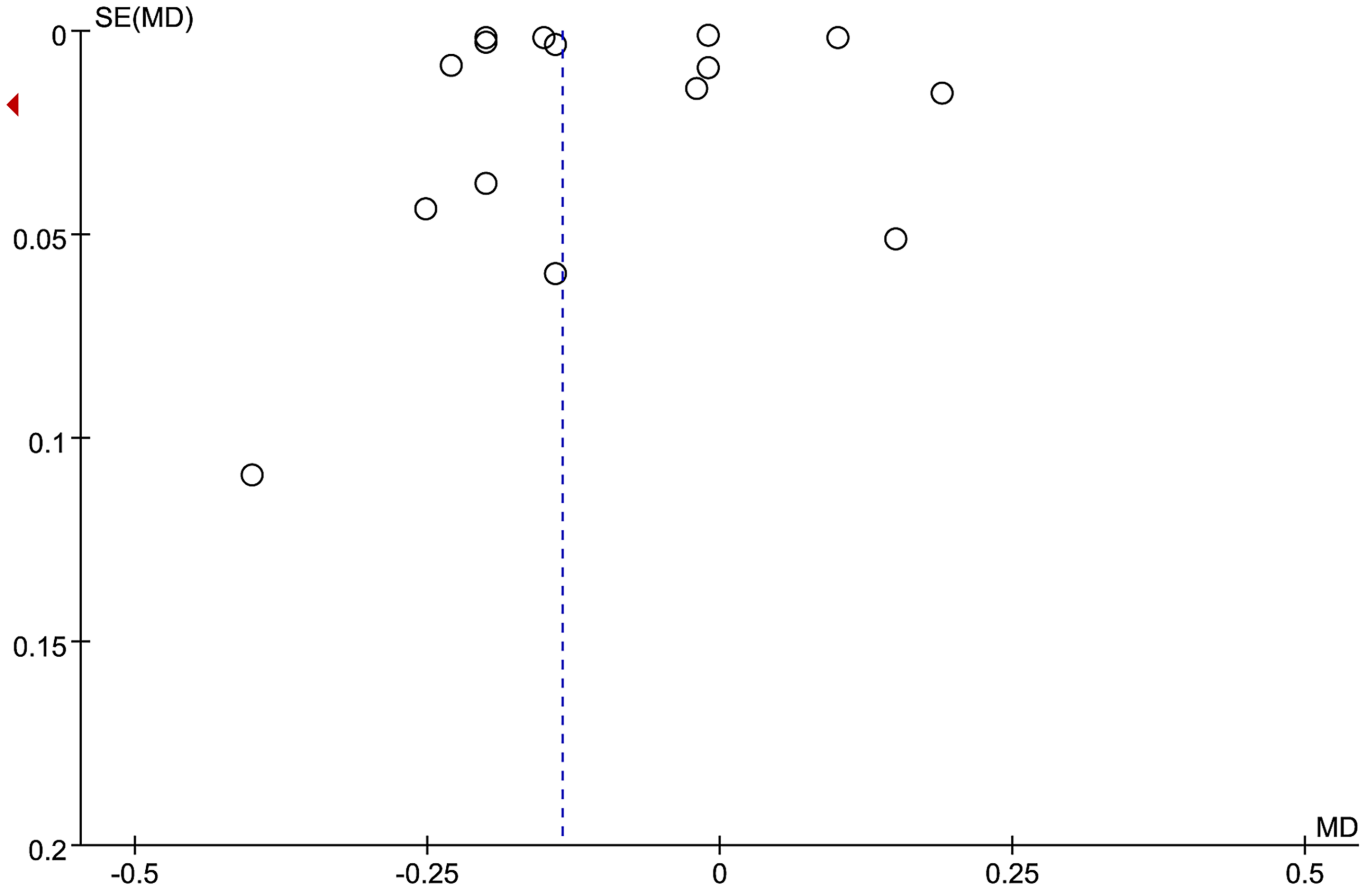
Funnel plot.

Visual inspection of the funnel plot revealed symmetry, indicating minimal publication bias (Figure 8).

## Discussion

Our meta-analysis revealed that circulating selenium levels were lower in ischemic stroke than controls. Meanwhile, we found significant inverse association between circulating selenium concentrations and stroke incidence as well as stroke mortality, indicating that the level of circulating selenium may play a role in the development of ischemic stroke. Participants with higher dietary selenium intake had a lower prevalence of stroke compared to the lower ones. Our dose–response meta-analysis revealed a significant inverse linear relationship between circulating selenium levels and ischemic stroke.

Selenium is an essential trace element that acts as a co-factor in various enzymes involved in key biological processes, including enzymatic antioxidant defense and immune system regulation. Some studies suggest that selenium deficiency may be associated with an increased risk of atherosclerotic cardiovascular disease (26) and atrial fibrillation (27). However, findings regarding the relationship between selenium and cerebrovascular disease remain inconsistent. Previous investigations (28–33) have extensively examined the association between circulating selenium concentrations and ischemic stroke. Mirończuk et al. (28) revealed that the serum selenium concentrations were significantly decreased in patients with ischemic stroke compared with healthy controls. Lower serum selenium levels were observed among acute ischemic stroke patients in Angelova et al. (29) study. The study by Nahan et al. (30) demonstrated that patients with acute ischemic stroke had markedly reduced circulating selenium concentrations versus controls. A similar finding was reported in Wang et al.’s study (31). The study by Kayama and colleagues (32) demonstrated that whole blood selenium concentrations were markedly reduced in patients with acute ischemic stroke patients relative to healthy controls. In contrast, study conducted by Skalny et al. (33) reported elevated circulating selenium levels in patients with acute ischemic stroke. Our present meta-analysis showed that circulating selenium levels were lower in ischemic stroke than controls, which was consistent with Ding and Zhang (20) study. Our meta-analysis demonstrated high heterogeneity, prompting subgroup analysis by study population. These analyses demonstrated significantly lower circulating selenium levels in ischemic stroke patients versus controls in non-Chinese populations, whereas no significant difference was observed in Chinese cohorts. These findings suggest that population-specific and regional factors may account for the substantial heterogeneity. The observed phenomenon may be attributed to both regional variations in selenium intake and differences in selenium metabolism.

Meanwhile, several studies have investigated the association between circulating selenium levels and stroke risk, but yielded inconsistent conclusions. Kok et al. (34) and Hu et al. (35) studies have suggested a negative connection between circulating selenium concentrations and ischemic stroke. A nested case–control research conducted by Xiao et al. (36) discovered that elevated circulating selenium levels were substantially correlated with a decreased risk of hemorrhagic stroke, but not with ischemic stroke. Our team conducted subgroup analyses showed an inverse correlation between circulating selenium levels and the risk of ischemic stroke, but not with hemorrhagic stroke, which is consistent with Hu’s finding (35). Notably, given the substantial heterogeneity observed, we conducted subgroup analyses by study type. There was an inverse association between circulating selenium levels and stroke risk in prospective and case–control studies rather than cross-sectional studies. It is worth noting that the inverse association between circulating selenium levels and stroke risk, as reported in the systematic review by Ding and Zhang (20), was specifically observed in cross-sectional/case–control studies, as opposed to prospective cohort designs. This discrepancy likely occurs because cross-sectional studies measure selenium levels at an undetermined time relative to stroke onset. When selenium is measured after stroke occurrence, it may reflect post-stroke metabolic changes rather than serving as causative risk factor. In light of this evidence, the high degree of heterogeneity may be attributable to critical variations in study design.

Notably, the inverse association between selenium levels and stroke has been predominantly observed in populations with generally low selenium exposure (e.g., China and Canada), while this protective relationship is less evident in regions with inherently high selenium status, such as the United States. Future research should focus on investigating the association between varying levels of selenium exposure and stroke risk.

The relationship between selenium concentrations and stroke risk is complex. Potential underlying mechanisms may include: First, Selenium exhibits potent antioxidant activity by up regulating selenoproteins such as glutathione peroxidase (GPx) and thioredoxin reductase (TrxR), thereby attenuating oxidative stress and potentially reducing cerebral infarction risk (37, 38). Second, selenium can improve atherosclerotic plaque stability and vasomotor function while inhibiting platelet aggregation and thrombus development (39). Third, Se-induced mitochondrial dysfunction prevention may considerably contribute to reducing lipid peroxidation and restoring ATP(Adenosine Triphosphate) concentrations, hence reducing infarct volume following localized cerebral ischemia (40). Fourth, selenium inhibits activation of the NF-κB inflammatory signaling pathway, resulting in down regulation of pro-inflammatory cytokines and consequent reduction in both the incidence and progression of cerebral infarction (41). Collectively, the available evidence suggests that selenium could confer dual neuroprotective benefits through both direct antioxidant mechanisms and indirect anti-inflammatory pathways in stroke prevention.

It is noteworthy that variations in selenium biomarkers (e.g., plasma, whole blood, serum, and selenoprotein) used across studies to assess the selenium-stroke association, which may contribute to the inconsistent findings observed in the literature (42). Whole blood analysis served as the primary method for determining selenium status in the earlier selenium research, the prolonged half-life of whole blood selenium establish its utility as a reliable indicator of chronic selenium exposure status (42). The most clinically useful biomarkers for assessing selenium status are serum and plasma, while plasma selenium has a short half-life and is more sensitive to short-term variations, mainly indicating current exposure status, which differ fundamentally from whole blood selenium. Impaired selenoprotein P function adversely affects selenium homeostasis, attenuating its cytoprotective effects and consequently increasing susceptibility to stroke (42). Therefore, measurement of selenoprotein P is necessary. Inconsideration of the selenium biomarkers and substantial heterogeneity, we conducted subgroup analysis, which examined the correlation between different selenium biomarkers (plasma, whole blood, serum, and selenoprotein) and the occurrence of stroke (43–46). Our present meta-analysis revealed that increased selenoprotein P and plasma/serum levels were linked to a decreased risk of ischemic stroke rather than whole blood. In contrast, the study by Ding and Zhang (20) reported an inverse association between whole blood selenium and stroke, while no significant association was observed for serum/plasma selenium. Thus, the employment of inconsistent biomarkers across included studies contributed substantially to the observed methodological heterogeneity.

The current study also identified a substantial link between circulating Se levels and stroke mortality. A cohort study (47) discovered that reduced circulating selenium concentrations were linked to a higher risk of stroke related mortality, which was in agreement with our findings. In contrast, no association was observed between stroke mortality and serum selenium in Wei et al.’s study (48). While some studies suggest a U-shaped relationship between selenium levels and the risk of cardiovascular disease as well as other health outcomes (49), no toxic effects were observed in the study. However, elevated selenium intake has been linked to potential toxicity in other reports (13). Recommended daily intake of selenium varies across regions. In the United States, the Food and Nutrition Board (FNB) suggests an Estimated Average Requirement (EAR) of 45 g/day, a Recommended Dietary Allowance (RDA) of 55 μg/day, and a Tolerable Upper Intake Level (UL) of 400 μg/day for adults aged 19–50 (50). In the United Kingdom, the Reference Nutrient Intake (RNI) is set at 60 μg/day for adult women and 75 μg/day for lactating women and adult men (51). For Chinese adults, the corresponding EAR, RNI, and UL values are 50, 60, and 400 μg/day, respectively (52). Significant geographical differences exist in baseline selenium levels, with some populations exhibiting notably higher concentrations than others. For Americans with adequate selenium intake, supplementation may lead to toxic effects, whereas selenium-deficient populations in China are more likely to benefit from it. Thus, regional selenium status should be considered when evaluating its association with stroke (16). The precise mechanism behind the negative correlation between selenium and stroke mortality remains uncertain. However, research (41) has indicated that selenium may provide benefits to stroke patients by decreasing the size of infarcts, enhancing prognosis, and reducing mortality rates.

Previous research have yielded conflicting findings on the significance of dietary selenium consumption in ischemic stroke (35, 44, 53, 54). A study (53) among Inuit in China revealed no link relationship between selenium consumption in the diet and the likelihood of having a stroke in persons with anemia. Conversely, a Canadian study (35) observed a reverse association between dietary selenium intake and prevalence of stroke in Inuit who exposed to high selenium levels through their traditional diet, consistent findings were reported in Chen et al.’s (54) study. Our study revealed an inverse connection between dietary selenium intake and stroke risk, indicating that elevated dietary selenium intake was associated with a decreased risk of stroke, which was consistent with Canadian’s study. To our knowledge, this is the first systematic review specifically investigating the relationship between dietary selenium intake and stroke incidence. As an essential trace element in antioxidant systems, selenium demonstrates significant cerebrovascular benefits, showing both prophylactic effects against stroke incidence and therapeutic potential for post-stroke recovery.

In our dose–response meta-analysis, a negative linear correlation was observed between dietary selenium intake and stroke risk. However, no definitive intake threshold was established for optimal protective benefit against stroke. These findings are partially inconsistent with extensive observational studies that suggest a potential L-shaped or non-linear relationship between selenium intake and ischemic stroke outcomes (55). Hu et al. (35) found that dietary selenium are reversely associated with the prevalence of stroke in Inuit, which follows an L-shaped relationship, and the estimated turning points of the L-shaped curve for dietary selenium was 350 μg/day. Meanwhile, nonlinear L-shaped this relationship was observed in the study by Zhang et al. (56) in Chinese, the cut-off point for selenium was 60 μg/day. In the study conducted by Shi et al. (53), dietary selenium intake had a negative and non-linear correlation with the risk of stroke in U.S. adults, with a nodal point observed at 105 μg/day beyond which no additional benefit was detected. Potential explanations for the observed discrepancies in findings may include: (1) Substantial geographic variations in dietary selenium intake: Regions like Venezuela, Canada, the USA, and Japan typically demonstrate high selenium intake, while European countries show significantly lower levels. Chinese populations exhibit even lower selenium intake, with most areas being selenium-deficient. Current evidence (54) generally supports a U-shaped relationship between selenium and health outcomes, mostly derived from all-cause mortality, with recognized toxicity at high exposure levels. The relationship between selenium and stroke may differ. We hypothesize that populations chronically exposed to high selenium levels may develop adaptive mechanisms, requiring greater selenium intake to maintain optimal selenoprotein activity, thereby exhibiting different dose–response patterns. (2) Population-specific characteristics in our analysis: Our systematic review primarily incorporated dose–response studies conducted in China—a typically low-selenium region where exposure levels rarely reach the toxic threshold (57). This may explain the observed linear inverse association in our analysis, contrasting with the U-shaped relationships reported in high-selenium populations.

The strengths of this meta-analysis are threefold. First, it represents the first comprehensive synthesis of evidence regarding the association between circulating selenium levels, dietary selenium intake, and stroke risk, supported by an extensive and systematic literature search. Second, the analysis relied on adjusted effect estimates from large-sample studies, enhancing the reliability of the findings. Third, potential heterogeneity was rigorously evaluated using established statistical methods, and where significant heterogeneity was identified, subgroup analyses and meta-regression were conducted to investigate underlying sources. The limitations of the current investigation should also be recognized. First, dietary selenium intake was self-reported in most studies, which may be influenced by recall bias, misclassification, as well as possible sources of confounding such as lifestyle variables, meanwhile, few studies adjusted for confounding dietary factors. Second, heterogeneous exposure categorization methods between studies likely contributed to measurement variability. Third, the lack of stroke subtype stratification precludes mechanistic insights into selenium’s potential differential effects. Fourth, the bio availability of selenium varies substantially across food sources, yet no studies examined source-specific effects on stroke risk—a critical gap for future research. Finally, most of the studies were from China, Canada and America, which may have limited applicability to populations with varying baseline selenium status, which highlight the need for multinational studies to validate these associations across populations with differing selenium statuses.

## Conclusion

Our meta-analysis discovered that circulating selenium concentrations are inversely associated with stroke risk and mortality, which suggesting its potential role as modifiable stroke risk factor. Meanwhile, higher dietary selenium intake shows an inverse correlation with ischemic stroke risk. Additional well-structured prospective cohort studies with precise selenium biomarker specifications and suitable selenium intake dosages are necessary to further elucidate these associations.
